# Single oral-dose fluralaner treatment against *Ophionyssus natricis* infestation – a large scale study demostrating long-term efficacy in captive snakes

**DOI:** 10.1007/s00436-026-08657-7

**Published:** 2026-03-07

**Authors:** Diego Cattarossi, Erica Marchiori, Federica Marcer, Paolo Zorzi, Elena Frara, Giulia Maria De Benedictis

**Affiliations:** 1 Veterinary Clinic “Casale sul Sile”, via Roma 47, Casale sul Sile (TV), 31032 Italy; 2 Department of Animal Medicine, Production and Health, University of Padova, viale dell’Università 16, Legnaro (PD), 35020 Italy; 3 Tropicarium Park, via Aquileia 123, Lido di Jesolo (VE), 30016 Italy

**Keywords:** Mites, Colubridae, Boidae, Treatment, Fluralaner

## Abstract

The hematophagous mite *Ophionyssus natricis*, vector of several viral and bacterial diseases in snakes, is one of the most challenging pathogens for snake breeders. The high environmental resistance of its larval stage makes repeated treatment of the animals unavoidable, while eradication of the mites from enclosures requires extensive cleansing and disinfection, often involving chemical products with known toxicity. The use of ectoparasiticides of the family of isoxazoline have recently proved effective against both adult and larvae of *O. natricis*, nevertheless data for fluralaner remains limited, particularly in large cohorts. In this study, 1579 snakes representing 18 different species were treated orally with a liquid formulation of fluralaner (Exzolt^®^) at 2.5 mg/kg, administered either by injection into the prey prior to feeding or via gastric gavage. Mite detachment and mortality occurred within 5 h, and no reinfestation was observed during subsequent 6-months follow-up period, despite the absence of environmental treatments. No side effects were reported in any treated animals, and no negative impact was observed on oviposition or hatching rate. The prolonged efficacy observed in this study further supports its suitability as a minimally invasive yet highly effective treatment of *O. natricis* infestations in snakes, given the long half-life of fluralaner already documented in mammals.

**Supplementary Information:**

The online version contains supplementary material available at 10.1007/s00436-026-08657-7.

## Introduction

Captive snakes are kept in a variety of contexts, including as domestic pets, in zoological collections, wildlife parks, mobile animal shows, and research facilities. Proper care and husbandry remain the cornerstone of reptile health in captivity; however, there is a lack of empirical data on how current practices affect snake welfare outcomes. This gap has important implications, as a better understanding of reptile management could inform initiatives aimed at improving animal welfare, including husbandry protocols and consumer education (Hutchings and Crane 2025; Wilkinson 2015). Among the many health challenges faced by captive snakes, ectoparasitic infestations represent a significant concern, often exacerbated by suboptimal environmental conditions. Parasitic diseases may represent a challenge to face, especially in densely-populated collections (Cooper 2006; Greiner and Mader 2006). Among them, infections by the hematophagous mite *Ophionyssus natricis* (Mesostigmata: Macronyssidae) represent one of most widespread and persistent problem encountered by reptile breeders. The remarkable resistance of the mite to unfavourable environmental conditions and its short life cycle (7–14 days) (Orlova et al. 2024), make this parasite exceedingly difficult to eradicate from captive settings, where mites can rapidly spread between enclosures (Rossi 2006).

Snake mites are highly mobile, allowing them to rapidly infest terrariums or enclosures (Wozniak and DeNardo 2000; Castro et al. 2019). Mite bites are painful, and severe infestations can cause significant distress to the host, resulting in dermatitis, difficulty or delay in ecdysis (shedding of skin), behavioural changes (such as prolonged immersion in water bowls or constant movement within the enclosure), and even death (Alfonso-Toledo and Paredes-León 2021; Hoppman and Wilson 2007). Severe infestations can also damage the loreal pits of venomous snakes (Simonov and Zinchenko 2010).

Health issues related to *O. natricis* in snakes are both from its direct effect, including blood loss and skin irritation, and from its role as a mechanical vector of infectious agents. *Ophionyssus natricis* has been implicated in the transmission of *Aeromonas hydrophila*, the causative agent of hemorragic disease in reptiles (Camin 1948), and, most notably, it has an important role in the diffusion of Arenavirs, Ferlavirus, Paramyxovirus and other viral diseases (Marschang et al. 2021). Indeed, poor control of mite infestations has been linked to recent outbreaks of viral diseases in snake collections. Furthermore, it is important to note that *O. natricis* represents a public health concern due to its role as a vector for zoonotic pathogens, such as the aforementioned *Aeromonas hydrophila*, and because it has tested positive in molecular studies for *Rickettsia* spp. of the spotted fever group (Mendoza-Roldan et al. 2021a, b). In addition, *O. natricis* may exhibit non-specific feeding behaviour on other host species, including humans, causing dermatitis and increasing the risk of zoonotic transmission of the these pathogens (Schultz 1975; Amanatfard et al. 2014; Šlapeta et al. 2017).

Available treatments rely on a multi-faceted approach, targeting adult mites on infested hosts, as well as all developmental stages present in the environment, in order to eliminate sources of re-infestation. Control measures must be sustained over time, as the highly motile mites can survive in the environment for up to three months without a blood meal (Šlapeta et al. 2017; Wozniak and DeNardo 2000). Treatment of infected snakes is generally attempted through repeated administration of chemical compounds. However, several of these approaches pose toxicological risks to snakes - particularly volatile pyrethrins, pyrethroids, organophosphates and carbamates used in the environment, or improperly rinsed fipronil applied directly to the animals (Gibbons et al. 2015; Orlova et al. 2024). Non-pharmacological methods include environmental modifications, such as raising the ambient temperature above 50° C and lowering humidity below 50%, both of which are detrimental to mite survival, as well as thorough disinfection and cleaning of enclosures, repeated warm-water baths for infested animals, and strict isolation or quarantine measures (Wozniak and DeNardo 2000). All of these treatment strategies are widely used in clinical practice, although no controlled trials have demonstrated their efficacy. In addition, their efficacy appears to have declined significantly over time, often requiring the combination of multiple protocols and frequent repetition of treatments to keep infestations under control (Cattarossi D, *pers. comm*.).

Fluralaner is an antiparasitic drug registered for its efficacy against ectoparasites, including fleas and ticks in domestic dogs and cats (Jiang and Old 2025). It is commercially available as Bravecto^®^ (Merck&Co., Inc., Rahway, NJ 07065, USA), in both oral and spot-on formulations. A notable advantage of fluralaner is its prolonged efficacy against target arthropods following a single administration. Similarly, fluralaner is approved for use in chickens (*Gallus gallus*) for the treatment of the red mite *Dermanyssus gallinae* (Mesostigmata: Dermanyssidae) (Petersen et al. 2021; Prohaczik et al. 2017). It is available as an oral formulation (Exzolt ^®^ 10 mg/ml, Merck & Co., Inc., Rahway, NJ 07065, USA) to be administered via drinking water at a dose of 0.5 mg/kg given twice at one week interval. This regimen targets two life cycles duration for *D. gallinae* and provides acaricidal efficacy for at least 15 days following treatment (Prohaczik et al. 2017).

Recent studies have investigated the use of isoxazoline compounds such as afoxolaner for the treatment of *O. natricis* infestations in snakes, reporting safe and effective results in *Lampropeltis* and other Colubridae and Boidae species (Mendoza-Roldan et al. 2023, Fuantos Gámez et al. 2020). To date, only one published study has evaluated the efficacy of fluralaner in snakes, using a group of 20 ball pythons (*Python regius*) treated with 40 mg/kg via oral route. The study reported positive results in terms of mite control on the animals, without the need for additional environmental treatments (Gobble 2022). Given the long half-life of fluralaner after a single administration, this molecule seems a particularly attractive candidate for parasite control in reptiles, especially in breeding facilities and zoological collections where repeated treatments are often impractical and can cause significant stress to the animals. This study was designed to evaluate the efficacy of a single oral dose of fluralaner at 2,5 mg/kg, to eliminate and prevent infestations by *O. natricis* without the need for environmental treatments. Given the wide variety of snake species kept in captivity and their frequent exposure to mite infestations, a large cohort of snakes representing multiple genera and species was included. In addition to evaluating antiparasitic efficacy, the study considered the potential effects on reproductive parameters, including oviposition, hatching success and neonatal health in breeding individuals.

## Materials and methods

A preliminary study was conducted at a private collection (Boa Line, Istrana, 31036 TV, Italy; *facility1*), including a limited number of individuals (*n* = 30): 10 Boas (*Boa constrictor imperator*), 10 Pythons (*Python regius*) and 10 Colubrids (*Pantherophis guttatus*, *Lampropeltis triangulum*). Based on the results of the preliminary trial, two larger groups of snakes were included in the study, namely 1,520 from the same collection (*facility1*) and 23 from a zoological collection (Tropicarium Park, 30016 Lido di Jesolo, VE, Italy, *facility2*). The list of species involved in this study is reported in Table 1. In both facilities, snakes were housed either individually or in small groups of no more than 2–3 individuals per enclosure. The animals ranged in age from 40 days to 14 years and body weight varied between 0.025 and 47.00 kg. The two facilities differed significantly in their husbandry conditions: *facility1* housed snakes in plastic boxes, with simple disposable substrate, whereas *facility2* housed animals in naturalistic exhibits with sandy substrate and environmental enrichment elements such as wood, rocks and vegetation.

**Table 1:** List of animals involved in the study per each species in the two facilities

Facility 1			Facility 2	
Boidae			Boidae	
*Boa constrictor imperator*	723		*Sanzinia madagascariensis*	2
*Corallus hortulana*	39		*Boa constrictor imperator*	5
Pythonidae			Pythonidae	
*Python regius*	371		*Python regius*	10
*Python molurus bivittatus*	4		*Python molurus bivittatus*	1
			*Python curtus*	1
			*Morelia viridis*	1
Colubridae			Colubridae	
*Hydrodynastes gigas*	33		*Zamenis longissimus*	1
*Pantherophis guttatus*	231		*Elaphe scalaris*	1
*Pantherophis obsoletus*	18		*Natrix maura*	1
*Lampropeltis triangulum hondurensis*	15			
*Lampropeltis triangulum nelsoni*	28			
*Heterodon nasicus*	37			
*Xenodon pulcher*	7			
*Pituophis melanoleucus lodingi*	12			
*Boiga dendrophila*	32			

**Fig. 1:**
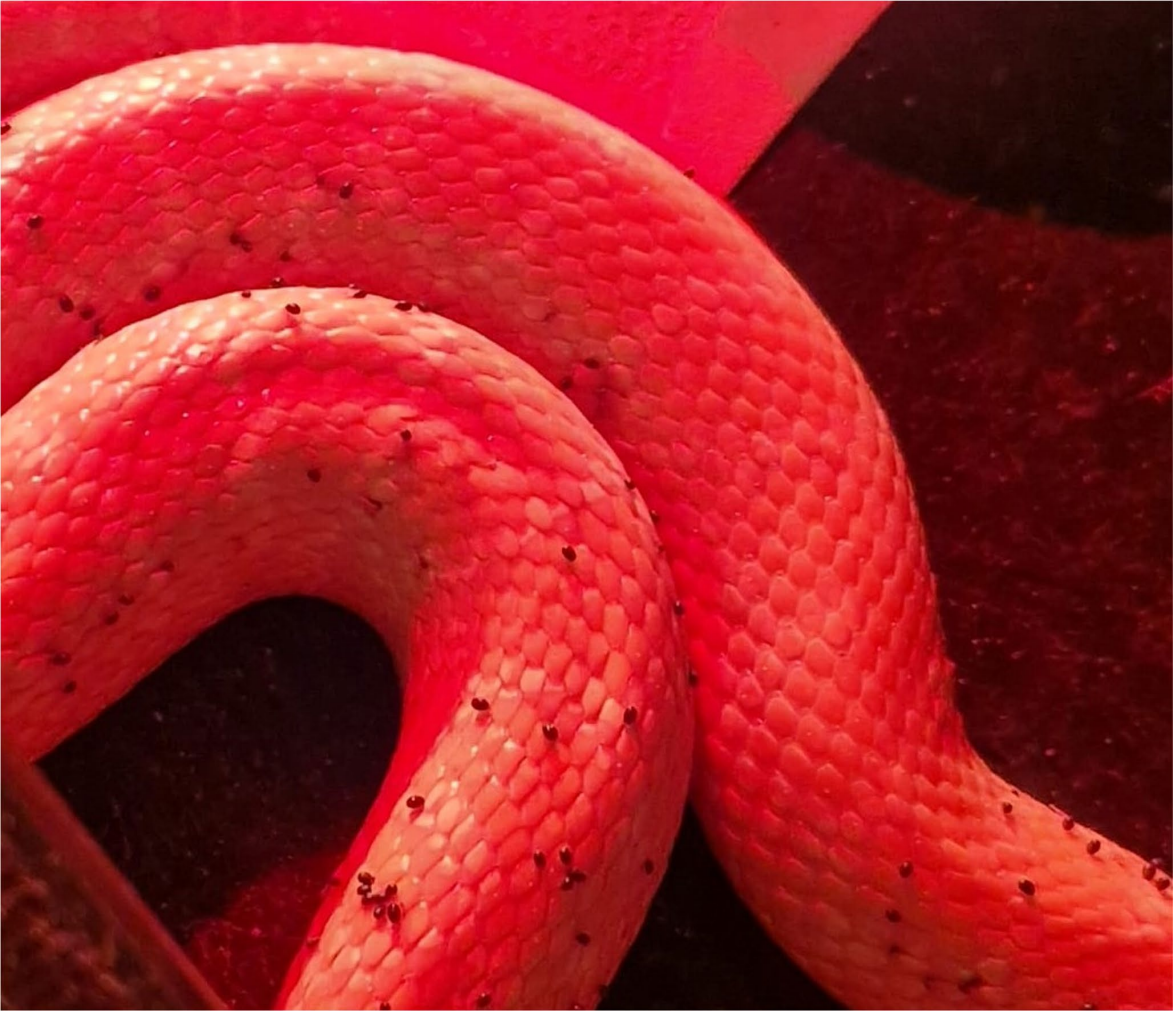
Adults of *O. natricis* feeding during night time on a *Boa constrictor* (infrared observation)

The clinical history provided by the owners of the facilities and a full clinical examination of all the animals, performed by the same veterinarian specialized in exotic animals, confirmed the presence of visible mites on 1,573 individuals including both facilities, even during daytime, contrasting the general tendency of the mites to feed during nocturnal hours (Fig. 1). Infestation level was evaluated on a semi-quantitative basis as mild, moderate, or severe, taking into consideration the presence and abundance of parasites in preferred microhabitats on the snakes’ body, namely peri-ocular area, gular area, lateral anterior and posterior scales, ventral anterior scales (Mendoza-Roldan et al. 2020). Individuals with severe parasitic burden were also found to show signs of physical distress and dermatitis.

Mites specimens (*n* = 20) were collected from infested individuals and submitted to species identification at the Laboratory of Parasitology of the Department of Animal Medicine, Production and Health of the University of Padova (Legnaro, PD, Italy). Mites were mounted in KOH and observed at the optic microscope and morphologically identified following keys in literature by Moraza et al. (2009). Molecular identification was also attained through amplification and sequencing of the rDNA small subunit (18 S) with the primer pair MITE18S_F and MITE18S_R (Otto and Wilson 2001). PCR products were visualized after electrophoresis in a 2% agar gel and positive samples were purified with ExoSap and sequenced at Macrogen^®^ (Macrogen Europe, Amsterdam, the Netherlands). Consensus sequences were compared with the non-redundant public database GenBank using the software BLAST^®^ (Sayers et al. 2025).

After clinical assessment, mite-infested animals were divided into groups of 20 animals and treated almost simultaneously, to facilitate the observations in the following hours. All animals were weighted and fluralaner (Exzolt^®^, 10 mg/ml) was administered orally at a dose of 2.5 mg/kg. The calculated dose was injected via graduated syringe into thawed prey (rat or mouse) shortly before feeding. Complete prey ingestion by the snake was checked after administration. In anorexic animals (*n* = 55), the drug was administered via oral gavage, by delivering a smaller prey, previously injected with the drug, into the distal oral cavity or esophagus, and then manually advanced to the stomach. Cages were monitored hourly for up to 5 h post treatment to assess both clinical status of the snakes and presence of dead mites on the cages bottom. At 5 h post-treatment, randomly chosen individuals - no less than 5 per treatment group - were handled to quantify mites on snake’s body. For this aim, selected skin areas representing preferred niches or microhabitats for *O. natricis* were carefully observed. Using the same clinical monitoring approach standard, animals were observed daily by the facility owners. and weekly by the specialist veterinarian for a period of up to six months post treatment. Evidence of resistant infestation or re-infestation was defined as visual detection of any single mite moving or firmly attached on snake’s body. No special environmental treatments were performed for sanitation, apart from weekly substrate removal in *facility 1*.

Oviposition was monitored by counting the number of produced eggs per clutch per each female in the six months following treatment, and percentage of hatching or of live newborns in viviparous species.

All data were analysed descriptively. No inferential statistical tests were performed, as the study design was observational and all animals achieved complete resolution.

## Results

Dead, immobile mites were observed on the cage bottom one hour after administration of fluralaner via oral gavage, and 3 h after treatment with injected prey. At the observation at 5 h post-treatment, no mites were visible on the targeted skin areas in none of the observed individuals. No other treatments were necessary in the following six months, as no mites were visible during daily and weekly clinical monitoring.

**Fig. 2:**
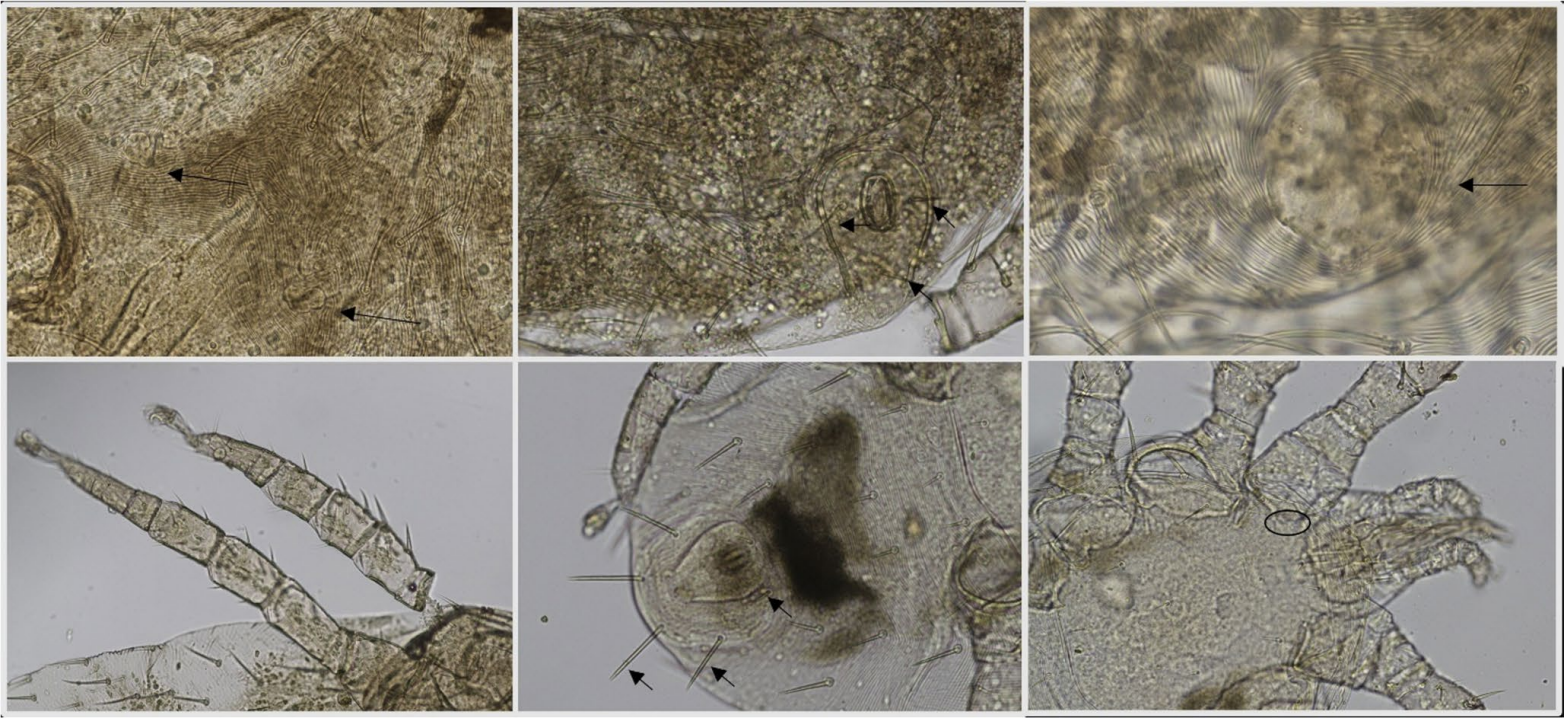
Photomicrographs of *Ophionyssus natricis* from this study. (**a**) Female, mesonotal scutellae (black arrow) (**b**) Female, anal shield showing three setae (black arrow) (**c**) Female, minute pygidial shield without setae (**d**) Male, absence of ventral spur in femur III (**e**) protonymoh with three pairs of setae on the pygidial shield (**f**) podonotal shield with setae z2 (black circle) (Moraza et al. 2009)

Morphological observation of the mites allowed identification of all specimens as *O. natricis* (Fig. 2). A sequence of 390 bp was obtained which blasted for 98.2% with the sequence of *O. natricis* (MT163327) in BLAST analysis.

No alteration of the physiological behaviour and organic functions of the snakes was registered during this time. No alteration of oviposition was observed in breeding females (*n* = 85) nor in frequency neither in number of produced eggs or newborns per clutch; egg hatching success remained similar to the previously registered rate in both facilities. No malformation was registered neither in oviparous nor in ovoviviparous species.

## Discussion

To our knowledge, no large-scale studies have evaluated the use of fluralaner for the treatment of mite infestation in snakes, despite the fact that this parasitic condition is a significant concern for reptile health and welfare. Ectoparasite infestations, particularly with O. *natricis*, are not only detrimental to the physical condition of the animals, but also complicate husbandry practices and biosecurity in captive environments. Commonly used chemical treatments in clinical practice include repeated applications of fipronil spray, pyrethrins and pyrethroids such as permethrin often mixed in spray formulation, organophosphates as trichlorphon (Fitzgerald and Vera 2006). Nevertheless, environmental, animal and human toxicity of most of these chemical compounds complicates treatment implementation, requiring careful dosing and close monitoring of the animals after treatment. Pyrethrins and pyrethroids must be rinsed immediately after application to avoid transcutaneous absorption and neurological signs (DeNardo and Wozniak 1997). Trichlorphon also acts neurotoxically; dosages must be accurately calculated avoiding solutions with concentration higher than 0.15%, and animals, especially more sensitive species, must be closely monitored for the onset of rigid paralysis after treatment (Fitzgerald and Vera 2006). Toxicity of all these compounds to the owner and other domestic animals must be taken into consideration, and the use of gloves and proper room ventilation is recommended when handling these products (Fitzgerald and Vera 2006). Ivermectin wether sprayed or injected seem to be safe in most species, and has the additional advantage of a relatively long biological half-life, remaining active in snake bood for extended periods, thereby making environmental treatment less essential (DeNardo and Wozniak 1997). Conversely, inefficacy has been reported at the recommended dosage (0.2 mg/kg) against other hematophagous mites (Ash and Oliver 1989).

Previous results on the use of isoxazolines have provided promising evidence supporting the availability of a safe, effective therapy, with monthly treatments (Mendoza-Roldan et al. 2023; Gobble 2022; Fuantos Gámez et al. 2020). Nevertheless, evidence is still limited to a small number of species and a relatively short period of observation after treatment. The present study is the first to evaluate the clinical efficacy of fluralaner in a large cohort of snakes, including a wide range of species housed in high-density environments. By demonstrating the efficacy of a single oral-dose protocol, this work provides promising evidence for a practical and welfare-oriented approach to mite control in both private breeding facilities and zoological collections. Rapid elimination of the mites was achieved, and no reinfestation occurred in the subsequent six months after administration, even in the absence of environmental treatments. This outcome is particularly notable given the known resilience of *O. natricis* and the complexity of mite control in high-density or enriched environments, such as zoological exhibits. A precise quantitative assessment of baseline infestation and complete clearance was not feasible due to practical constraints, such as counting all mites—including smaller, less visible stages—across such a large cohort. Nevertheless, a semi-quantitative evaluation was applied to verify treatment efficacy, including in the most severely affected animals. The long-term absence of clinical signs or visible mites in any treated specimens provides strong evidence of effectiveness. The absence of a control group may limit the interpretation of the result. Nevertheless, both facilities had a history of persistent infestation, with multiple treatments previously attempted without a definitive resolution, resulting in recurrently infestation over short period. Environmental and husbandry conditions during those previous attempts were identical to those applied in this study. For this reason, and in consideration of animal welfare, formal control groups were not settled; however, the prior ineffective treatment attempts can be regarded as a reference for comparison. Finally, infestation was evaluated only by visual detection, so the presence of smaller, early parasitic stages on the snakes at the time of examination cannot be excluded. This is most likely during the first period after treatment, as environmental stages of the parasites were not directly affected by the treatment, and could survive until reaching the parasitic stage. Contact with the snakes’ skin and blood, however, likely exposed the mites to the drug, resulting in their death and preventing development into later stages, as no adult mites were subsequently observed. Nevertheless, further studies focusing on the detection of early parasitic stages – deutonymphs - on the snakes and the timing of their survival may be recommended to address these points.

Although no pharmacokinetic tests were performed in this study, the clinical results are consistent with prior findings on isoxazolines. Afoxolaner has been shown to maintain effective plasma concentrations for up to 28 days after dosing in snakes, mirroring data from mammals (Mendoza-Roldan et al. 2023). Among other isoxazolines, fluralaner is recognised for its extended half-life and long-lasting efficacy in treating and preventing ectoparasite infestations in cats and dogs and chickens (Kilp et al. 2014; Sun et al. 2025). Its activity lasts up to 12 weeks in the studied species, likely explains the prolonged efficacy observed against snake mite infestations, which is probably due to sustained plasma concentrations sufficient to interrupt the entire life cycle of snake mites. Furthermore, its mechanism of action involves selective inhibition of gamma-aminobutyric acid (GABA)-gated chloride channels in arthropods, while its low affinity for vertebrate GABA receptors ensures a wide margin of safety in mammals and birds (Ozoe and Asahi 2025; Sun et al. 2025). Further pharmacokinetic studies in different snake species would be useful to confirm this hypothesis and optimize dosing protocols. The off-label use of fluralaner in this study was based on a preliminary trial conducted in a limited number of animals, in which no adverse effects were observed. Moreover, the dose administered in the present study was lower than that previously used by Gobble (2022). In Italy, no antiparasitic drugs are currently registered for use in snake species; therefore, any treatment of snake infestations must be performed using products registered for other species.

The dosage of 2.5 mg/kg used in this study was extrapolated from data reported in mammalian studies (Kilp et al. 2014). Although significantly lower than the 40 mg/kg dose previously described in literature for pythons (Gobble 2022), this dosage proved sufficient to achieve mite eradication in all treated snakes. Notably, fluralaner is also registered for use in chickens (Exzolt^®^) for the control of red mites where two doses of 0.5 mg/kg administered one week apart are recommended to achieve complete clearance (Petersen et al. 2021). Further studies are encouraged to assess pharmacokinetics of fluralaner in snakes, in order to determine the minimum effective dose to obtain significant suppression of mite numbers, encompassing the period of resistance of non-feeding, free-living larval stages - approximately 31 d for deutonymphs (Wozniak and DeNardo 2000). In this study, the efficacy of the drug after single administration was long enough to overcome deutonymphs resistance, without the need of environmental treatments - even in the zoological park where enclosure enrichments in the snakes cages offered numerous suitable microhabitats for mites survival. Experimental conditions were not standardized in this study between the two facilities, which may represent a limitation for accurate evaluation of environmental factors eventually affecting treatment efficacy. Moreover, observation of dead mites in the cage internals was much more difficult in the enriched enclosures of *facility 2*, due to different, irregular and darker substrate compared to the cages of *facility 1*. Nevertheless, no reinfestation was observed in the following six months in animals kept in enriched exhibits, finally highlighting the potential for wide use of this drug in many different captivity contexts.

Fluralaner was already tested for its efficacy in ball pythons (*Python regius*) (Gobble et al. 2022), through use of gastric probes starting from chewable tablets and attaining a proper dilution with water. The liquid formulation used in this study overcome the need for drug preparation and offers a reliable calculation of the dosage administrated to the snake. Furthermore, the possibility of administering the drug through injected preys permits to avoid the stressful event of forced feeding and increase the easiness of administration and consequently breeders’ compliance. No side effects, such as regurgitation, were observed in any animal. Observation of a high number of individuals belonging to different families allowed to test widely the safety of this treatment off-label including small and big sized animals.

## Supplementary Information

Below is the link to the electronic supplementary material.

Supplementary figure 1 Electrophoresis gel of amplified 18S portion after PCR (Otto and Wilson 2001) (DNA ladder EuroClone SHARPMASS ^TM^ 100). For each template two different DNA concentrations were used. (PNG 2.04 MB)

High Resolution Image (TIF 596 KB)

## Data Availability

All data supporting the findings of this study are available within the paper and its Supplementary Information.
