# Supplementary figures and images for: Single oral-dose fluralaner treatment against Ophionyssus natricis infestation – a large scale study demostrating long-term efficacy in captive snakes
Source: Parasitol Res. 2026 Mar 7;125(1):36. doi: 10.1007/s00436-026-08657-7 (PMC12967550; doi:10.1007/s00436-026-08657-7)

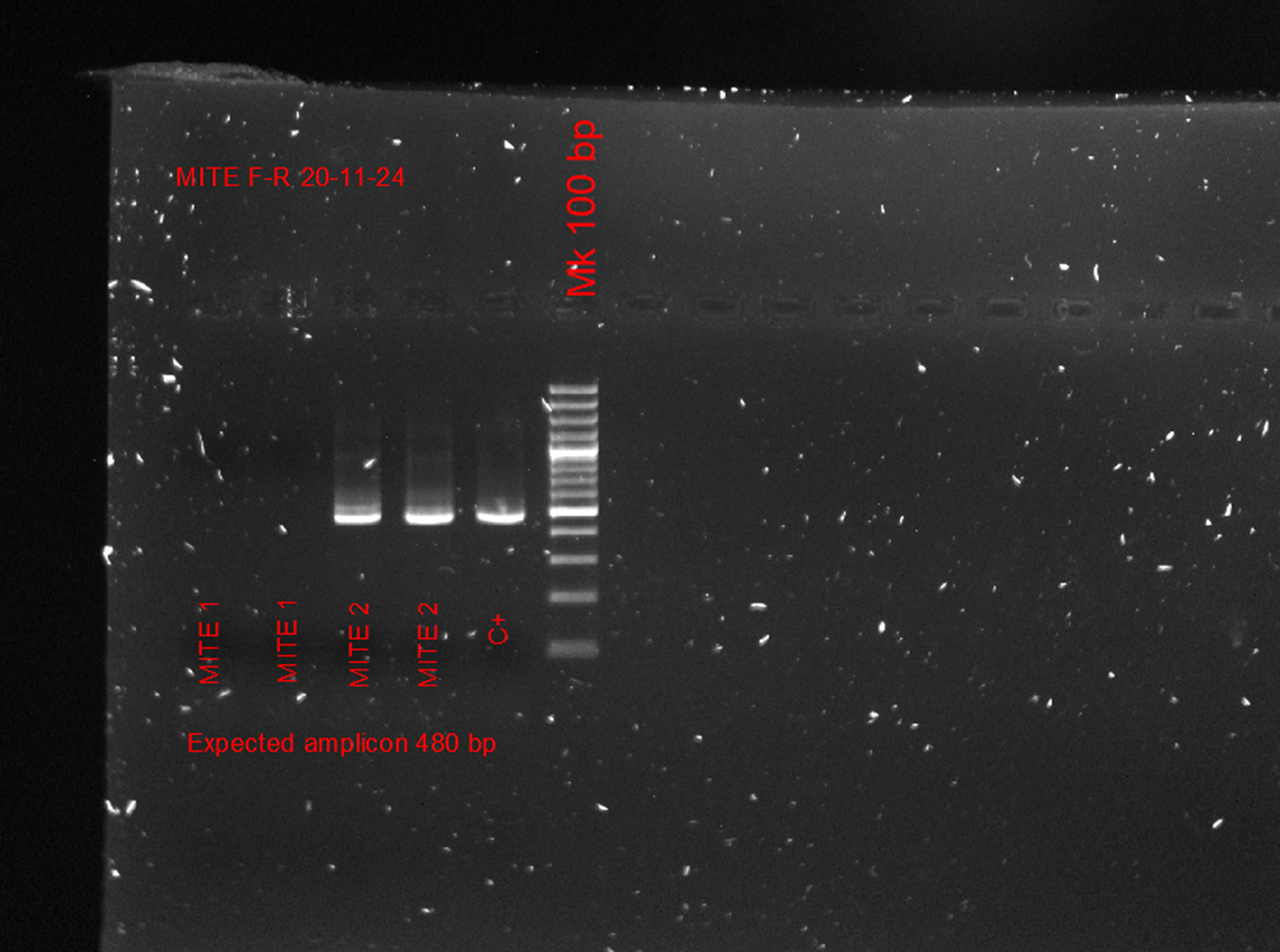

Supplement: Supplementary file 1 — Electrophoresis gel of amplified 18S portion after PCR (Otto and Wilson 2001) (DNA ladder EuroClone SHARPMASS TM 100). For each template two different DNA concentrations were used. (PNG 2.04 MB) [file 436_2026_8657_Fig3_ESM.png]

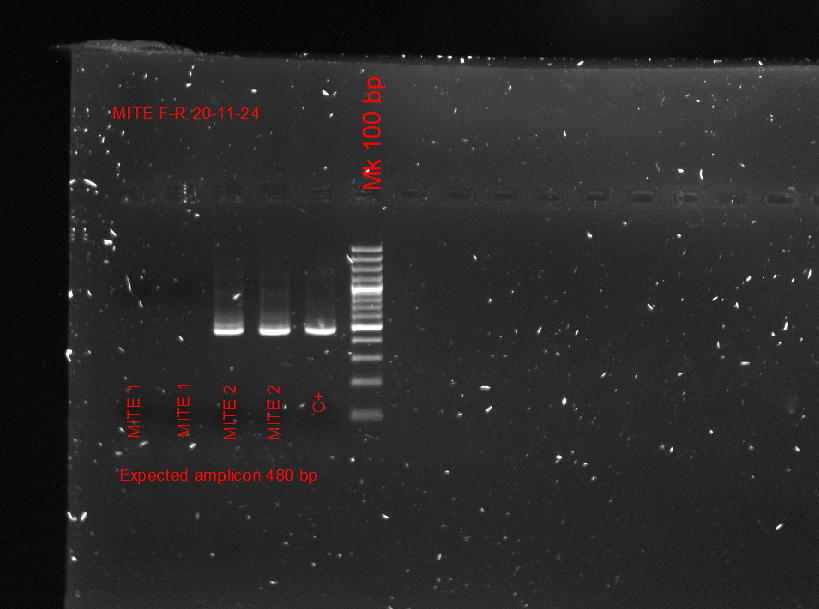

Supplement: Supplementary file 2 — High Resolution Image (TIF 596 KB) [file 436_2026_8657_MOESM1_ESM.tif]
